# Prognostic Value of Baseline High-Uptake PSMA Tumor Volume Fraction in Metastatic Castration-Resistant Prostate Cancer Patients Treated with [^177^Lu]Lu-PSMA Radioligand Therapy

**DOI:** 10.3390/cancers18203297

**Published:** 2026-10-13

**Authors:** Marco Cuzzocrea, Giovanna Pecoraro, Cesare Michele Iacovitti, Chiara Martinello, Giorgio Treglia, Silke Gillessen, Martino Pedrani, Gaetano Paone

**Affiliations:** 1 Clinic for Nuclear Medicine, Institute of Integrated Diagnostics of Southern Switzerland (IDISI), Ente Ospedaliero Cantonale (EOC), 6500 Bellinzona, Switzerland; cesaremichele.iacovitti@eoc.ch (C.M.I.); chiara.martinello@eoc.ch (C.M.); giorgio.treglia@eoc.ch (G.T.); gaetano.paone@eoc.ch (G.P.); 2 Department of Medical Oncology, Oncology Institute of Southern Switzerland (IOSI), Ente Ospedaliero Cantonale (EOC), 6500 Bellinzona, Switzerland; giovanna.pecoraro@eoc.ch (G.P.); silke.gillessensommer@eoc.ch (S.G.); martino.pedrani@eoc.ch (M.P.); 3 Institute of Oncology Research (IOR), 6500 Bellinzona, Switzerland; 4 Clinical and Translational Oncology (CTO), Scuola Superiore Meridionale, 80134 Naples, Italy; 5 Faculty of Biomedical Sciences, Università della Svizzera Italiana, 6900 Lugano, Switzerland; 6 Faculty of Biology and Medicine, University of Lausanne, 1015 Lausanne, Switzerland

**Keywords:** Lu-PSMA, mCRPC, PSMA-PET/CT, prognostic biomarker, theranostics

## Abstract

**Background/Objectives:** Lutetium-177 prostate-specific membrane antigen radioligand therapy ([^177^Lu]Lu-PSMA-RLT) is an effective treatment for patients with metastatic castration-resistant prostate cancer (mCRPC), but no validated PSMA-PET/CT-based biomarker currently identifies patients at risk of early treatment failure. The aim of this study was to assess whether semi-quantitative PSMA-PET/CT parameters, in particular the fraction of PSMA-positive tumor volume with high uptake, are associated with early biochemical progression (EBP) and PSA50 response and secondarily with PSA-PFS and OS. **Methods:** Semiquantitative PSMA-PET parameters comprised reference lesion SUVmax (RL-SUVmax), SUVmean, total PSMA tumor volume (tPSMA-TV), tPSMA-TV above a fixed SUV threshold of 20 (tPSMA-TV20), the percentage of tPSMA-TV20 within tPSMA-TV (tPSMA-TV%), total lesion quotient (TLQ), TLQ20, and rTLQ%. The co-primary endpoints were EBP, defined as PSA progression according to the PCWG3 criteria within 4 months of the start of treatment, and PSA50 response, defined as a reduction in PSA of at least 50% compared with baseline. PSA-PFS (PCWG3-defined PSA progression or death from any cause) and OS were secondary endpoints. **Results:** 63 patients were retrospectively included (median follow-up, 20.9 months). In univariable analyses, higher baseline tPSMA-TV% was significantly associated with lower odds of EBP (OR per 10% increase, 0.71; 95% CI, 0.57–0.87; *p* = 0.001) and higher odds of PSA50 response (OR, 1.31; 95% CI, 1.07–1.61; *p* = 0.009). It was associated with longer PSA-PFS (HR, 0.75; 95% CI, 0.67–0.84; *p* < 0.001) and longer OS (HR, 0.81; 95% CI, 0.72–0.91; *p* < 0.001). Exploratory ROC analysis identified a tPSMA-TV% cut-off of 47.54% for EBP (AUC, 0.73 [95% CI, 0.57–0.86]; sensitivity, 0.61; specificity, 0.93), although this threshold requires external validation. In exploratory models including tPSMA-TV and SUVmean, tPSMA-TV% remained associated with PSA-PFS and OS but not with EBP or PSA50 response. **Conclusions:** Baseline tPSMA-TV% was associated with EBP, PSA50 response, PSA-PFS and OS in mCRPC patients undergoing [^177^Lu]Lu-PSMA-RLT, and these associations remained after adjustment for baseline PSA and metastatic presentation. tPSMA-TV% is a candidate prognostic imaging biomarker that requires external validation.

## Simple Summary

Lutetium-177 prostate-specific membrane antigen radioligand therapy is an effective option for metastatic castration-resistant prostate cancer, yet a substantial proportion of patients progress early and no validated PSMA-PET/CT biomarker is available to identify them before treatment. In 63 patients, we assessed semiquantitative baseline PSMA-PET/CT parameters describing the extent and distribution of PSMA-avid tumor burden, focusing on the proportion of total PSMA-positive tumor volume with uptake above a fixed SUV threshold of 20 (tPSMA-TV%). Higher baseline tPSMA-TV% was associated with lower odds of early biochemical progression and higher odds of PSA50 response and with longer PSA progression-free survival and overall survival; an exploratory cut-off of 47.54% discriminated early biochemical progression with high specificity but requires external validation. Because tPSMA-TV% is derived from the pretherapeutic scan already acquired in routine practice, it could contribute to baseline risk stratification before the first cycle, identifying patients at higher risk of early failure who may require closer monitoring.

## 1. Introduction

Metastatic castration-resistant prostate cancer (mCRPC), the most advanced stage of prostate cancer, is associated with limited survival benefit despite androgen deprivation therapy, androgen receptor pathway inhibitors, and docetaxel [1]. Lutetium-177 prostate-specific membrane antigen radioligand therapy ([^177^Lu]Lu-PSMA-RLT) improves outcomes compared with standard of care in patients with PSMA-positive disease without measurable PSMA-negative lesions, demonstrating significantly longer radiographic progression-free survival and OS in the VISION trial [2]. The TheraP trial [3] required high PSMA expression (SUVmax > 20) and additionally used [^18^F]FDG-PET/CT to exclude PSMA-discordant disease, resulting in higher PSA response rates than cabazitaxel, although without prolonging OS. Despite demonstrated survival and quality-of-life benefits [2,4,5], approximately one-third of patients show suboptimal response, with early biochemical progression (EBP) occurring within the first two treatment cycles [6], prompting interest in predictive and prognostic factors that could improve patient selection and outcomes.

Clinical and laboratory variables, including elevated baseline LDH and visceral metastases, are linked to reduced efficacy [7], while early PSA decline predicts favourable response and survival [8]. Semiquantitative PSMA-PET/CT metrics, including total PSMA tumor volume (tPSMA-TV), SUVmax/SUVmean, and composite burden indices, have shown correlations with OS, PFS, and treatment response [9,10,11,12,13,14]. Target expression is central to patient selection because the radiation dose delivered by [^177^Lu]Lu-PSMA depends on ligand uptake, as highlighted by the selection criteria for the VISION and, above all, TheraP studies [2,3]. However, few studies have evaluated whether the distribution of PSMA expression within tumor burden, rather than volume or SUV indices alone, predicts EBP, which may reflect intrinsic radioresistance or tumor heterogeneity [15]. Nomograms combining clinical and imaging parameters have also been externally validated for predicting response and survival [16], yet no standardised approach currently exists for selecting patients most likely to benefit from [^177^Lu]Lu-PSMA-RLT [17]. Moreover, evidence across clinical settings [18,19,20,21,22,23] and ongoing trials [24] may further support earlier treatment administration, potentially refining treatment algorithms.

This study aims to evaluate whether semiquantitative PSMA-PET/CT parameters reflecting the extent and intensity distribution of PSMA expression, assessed before [^177^Lu]Lu-PSMA-RLT, can identify patients at higher risk of EBP and lower likelihood of PSA50 response, and are secondarily associated with PSA-PFS and OS, thereby potentially helping to personalise treatment.

## 2. Materials and Methods

### 2.1. Patients and Study Design

This retrospective, single-centre observational study included consecutive mCRPC patients who started [^177^Lu]Lu-PSMA-RLT at our institution between July 2020 and March 2026 and who had a baseline PSMA PET/CT scan, completed at least one treatment cycle, and had sufficient follow-up data. The patient flow, including the reasons for exclusion, is shown in Supplementary Figure S6. Clinical variables collected included baseline PSA, prior treatments, liver involvement, and disease presentation. The study was conducted in accordance with the Declaration of Helsinki under a retrospective data-collection protocol approved by the Cantonal Ethics Committee of Ticino (protocol number 2023-01208); informed consent was handled according to the approved protocol, as detailed in the Informed Consent Statement.

### 2.2. Treatment

[^177^Lu]Lu-PSMA-RLT ([^177^Lu]Lu-PSMA-617 {Pluvicto^®^, lutetium [177Lu] vipivotide tetraxetan; Novartis Pharma Schweiz AG, Rotkreuz, Switzerland} or [^177^Lu]Lu-PSMA-I&T {Curium Finland, Helsinki, Finland}) was administered intravenously at 7.4 GBq (+/−5%) per cycle every 6–8 weeks following EANM guidelines [17], with standard premedication per institutional protocol. The radioligand used depended on institutional availability during the study period and on the decision of the physician based on the label of the compound in accordance with national legislation: [^177^Lu]Lu-PSMA-617 was used until 2022 and again from 2025, and [^177^Lu]Lu-PSMA-I&T mainly in 2023–2024. Up to six cycles were planned; treatment was discontinued at disease progression, unacceptable toxicity or patient decision.

### 2.3. PSMA PET/CT Acquisition and Analysis

**Figure 1:**
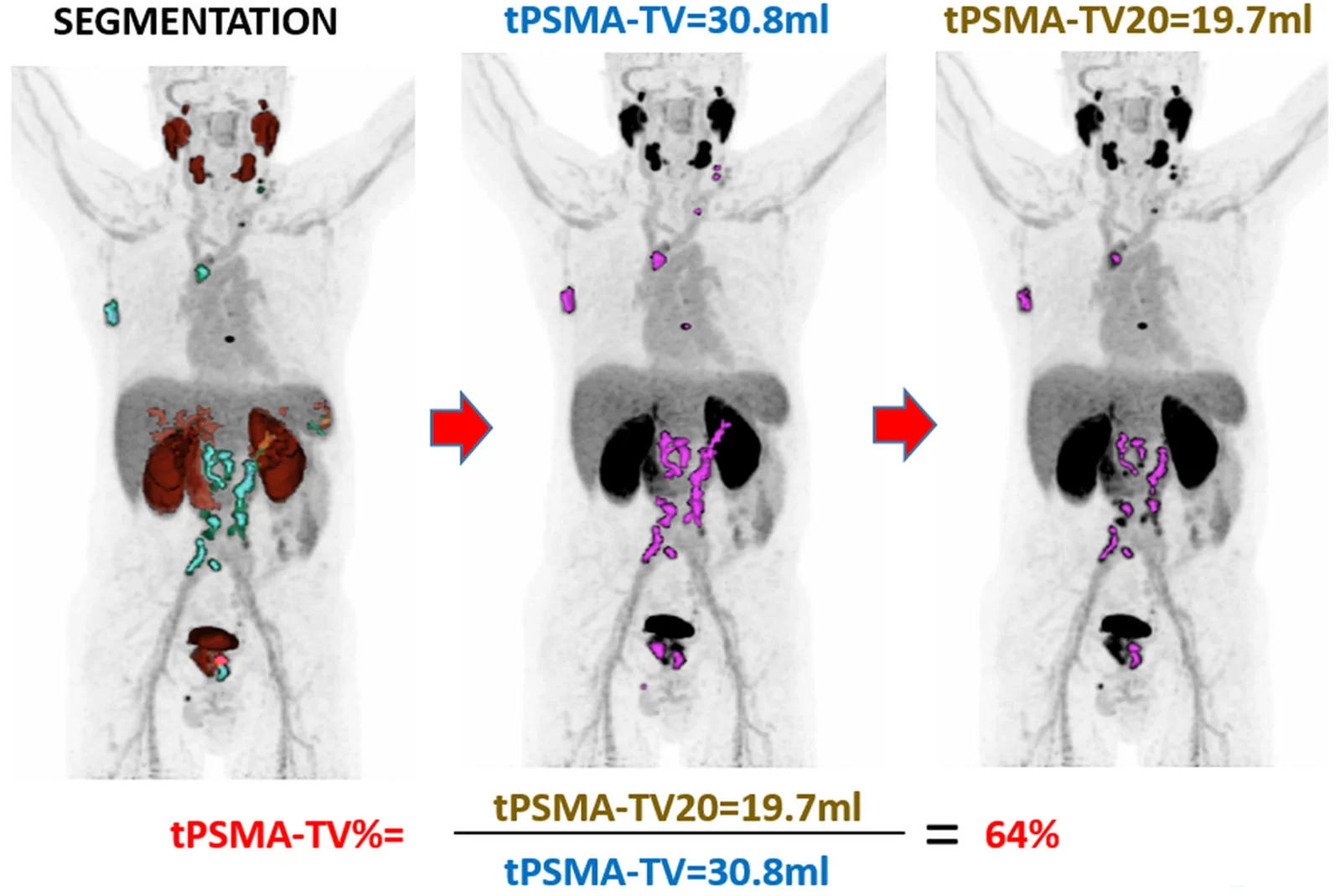
Representative PSMA PET segmentation workflow used to derive total PSMA-positive tumor volume (tPSMA-TV), high-uptake tumor volume above SUV 20 (tPSMA-TV20), and their ratio (tPSMA-TV%). tPSMA-TV20 is the volume of voxels with SUV ≥ 20 within the tumor volumes of interest.

PET/CT scans were acquired on a Biograph^®^ mCT40 (Siemens, Erlangen, Germany) 60 min after injection of [^68^Ga]Ga-PSMA-11 (n = 33) {kit for preparation of 68Ga-gozetotide injection, Locametz®, Novartis Pharma Schweiz AG, Rotkreuz, Switzerland} or [^18^F]F-PSMA-1007 (n = 30) {Center for Radiopharmacy, USZ, Zurich, Switzerland} at a mean administered activity of 3.7 MBq/kg [25]. Two nuclear medicine physicians blinded to clinical data performed image analysis using semi-automatic segmentation (MM Oncology, Lesion Scout, syngo.via^®^ v.8.0, Siemens) in consensus. Lesions detected by the software were verified by the readers; physiological uptake (lacrimal and salivary glands, liver, spleen, kidneys, bowel and urinary tract) was excluded, and volumes adjacent to physiologically avid organs or urinary activity were corrected manually by placing a manual VOI using the same segmentation criteria as for the other lesions. Each lesion was delineated with a volume of interest (VOI) using a threshold of 40% of the lesion SUVmax. Eight semiquantitative PET parameters were extracted. RL-SUVmax is the SUVmax of the reference lesion, defined as the lesion with the highest SUVmax. SUVmean is the mean SUV of all tumor VOIs. The total PSMA-positive tumor volume (tPSMA-TV, mL) is the sum of the volumes of all tumor VOIs. tPSMA-TV20 (mL) is the volume of the voxels within these VOIs with a SUV threshold of 20; it is therefore a voxel-wise sub-volume, not the volume of lesions whose SUVmax exceeds 20 (Figure 1). tPSMA-TV% = 100 × tPSMA-TV20/tPSMA-TV. The total lesion quotient (TLQ, SUV × mL), analogous to total lesion PSMA, is the product of SUVmean and tPSMA-TV; TLQ20 is the corresponding product within tPSMA-TV20, and rTLQ% = 100 × TLQ20/TLQ is the share of total tumor uptake contributed by the high-uptake volume. The SUV threshold of 20 was chosen a priori because it corresponds to the SUVmax required for PSMA-PET eligibility in the TheraP trial [3]; segmentation was performed at this threshold only. An example of semi-automatic segmentation is reported in Figure 1.

### 2.4. Endpoints and Statistical Analysis

The co-primary endpoints were EBP and PSA50 response; PSA-PFS and OS were secondary endpoints. Because no standardized terminology currently exists to describe PSA progression occurring very early after treatment initiation, we defined this outcome as EBP (PCWG3-defined PSA progression within 4 months of [^177^Lu]Lu-PSMA-RLT initiation) to capture patients who derive minimal or no benefit from treatment and in whom early identification could most directly inform treatment decisions. EBP was ascertained from the PSA values measured before each cycle and during follow-up; death without documented PSA progression was not counted as EBP, and patients alive without documented PSA progression were classified as not having EBP. PSA50 response was defined as a PSA decline of at least 50% from baseline. PSA-PFS was defined as the time from the first [^177^Lu]Lu-PSMA-RLT cycle to PCWG3-defined PSA progression or death from any cause, whichever occurred first [26]. Patients alive without PSA progression were censored at last available follow-up. OS was defined as the time from the first [^177^Lu]Lu-PSMA-RLT cycle to death from any cause or last follow-up. Median follow-up was estimated with the reverse Kaplan–Meier method. Cox regression was used for secondary time-to-event outcomes; logistic regression for primary dichotomous endpoints. The primary biomarker tPSMA-TV% was analysed as a continuous variable; an exploratory ROC-derived cut-off was identified via the Youden index. Multivariable analyses adjusted for log-transformed baseline PSA and de novo versus metachronous presentation. These covariates were selected a priori on clinical grounds and not by statistical selection: baseline PSA is an established prognostic factor after [^177^Lu]Lu-PSMA-RLT [16], and metastatic presentation reflects disease biology and prior treatment exposure. The adjustment set was limited to two covariates because of the number of events (23 EBP events). These models test whether the association of tPSMA-TV% is independent of selected clinical variables; they were not designed to demonstrate incremental performance over established PET parameters. Multiplicity was controlled with the Benjamini–Hochberg false discovery rate across the two co-primary endpoints and within each endpoint for the exploratory PET parameters (Supplementary Tables S1–S3). Additional exploratory analyses comprised Spearman correlations between PET parameters; the incremental value of tPSMA-TV% over log2-transformed tPSMA-TV and SUVmean, assessed with the likelihood ratio test and the change in the area under the ROC curve (AUC) or Harrell’s C-index with bootstrap 95% confidence intervals (1000 resamples); models adjusted for, and stratified by, PET tracer and therapeutic radioligand, with tPSMA-TV% × tracer and tPSMA-TV% × radioligand interaction terms; and sensitivity analyses of EBP excluding patients whose EBP status could not be ascertained and counting early deaths as events. Cox proportional hazards models used Breslow handling of ties. All tests were two-sided and *p* values < 0.05 were considered statistically significant. Analyses were performed using Python 3.12.13 with pandas 3.0.1, NumPy 2.3.5, statsmodels 0.14.6, lifelines 0.30.3 and scikit-learn 1.9.0; the additional Cox models used Efron handling of ties.

The detailed materials and methods are contained in the Supplementary Materials.

## 3. Results

Between July 2020 and March 2026, 82 patients were evaluated for [^177^Lu]Lu-PSMA-RLT; 19 did not start treatment (at least one PSMA-negative lesion on baseline PSMA-PET/CT, n = 16; rapid disease progression before PSMA-PET/CT, death, or refusal of treatment, n = 1 each), and the remaining 63 patients were included in the study (Supplementary Figure S6). Median age at baseline was 81 years (IQR, 72–84). De novo metastatic disease was present in 32 patients (50.8%), whereas 31 (49.2%) had metachronous metastatic disease. Median baseline PSA level was 42.62 ng/mL (IQR 8.36–216.50), and liver involvement was present in 14 patients (22.2%). Baseline PSMA-PET/CT was performed with [^68^Ga]Ga-PSMA-11 in 33 patients (52.4%) and with [^18^F]F-PSMA-1007 in 30 (47.6%); 36 patients (57.1%) received [^177^Lu]Lu-PSMA-617 and 27 (42.9%) [^177^Lu]Lu-PSMA-I&T. The median interval between baseline PSMA-PET/CT and initiation of [^177^Lu]Lu-PSMA-RLT was 41 days (IQR, 28–56). Patients received a median of 3 cycles (IQR, 2–4) of ^177^Lu-PSMA, with a median cumulative administered activity of 20.7 GBq (range 7–44.2). The median baseline tPSMA-TV% was 61.4% (IQR, 46.2–73.8). During follow-up, 51 patients (81.0%) experienced a composite PSA-PFS event, including 44 patients with documented PSA progression and 7 deaths before documented PSA progression; forty patients (63.5%) died. Median follow-up was 20.9 months (95% CI, 15.4–29.4); median PSA-PFS was 4.4 months (95% CI, 3.7–6.0) and median OS 13.8 months (95% CI, 9.9–15.5). EBP occurred in 23 patients (36.5%). Three patients died within 4 months without documented PSA progression and seven patients were alive without PSA progression after less than 4 months of PSA follow-up; these 10 patients were classified as not having EBP (sensitivity analyses in Section 3.4). A PSA50 response was observed in 30 patients (47.6%). Baseline characteristics of patients are summarized in Table 1.

### 3.1. Primary Endpoints

In univariable logistic regression (Table 2), higher baseline tPSMA-TV% was significantly associated with lower odds of experiencing EBP. For every 10% increase in tPSMA-TV%, the odds of EBP decreased by approximately 29% (OR 0.71, 95% CI 0.57–0.87; *p* = 0.001). Higher baseline tPSMA-TV% was also associated with a greater likelihood of PSA50 response (OR 1.31 per 10% increase, 95% CI 1.07–1.61; *p* = 0.009). Both associations remained significant after false discovery rate correction across the two co-primary endpoints (q = 0.002 and q = 0.009).

In multivariable logistic regression adjusted for log-transformed baseline PSA and de novo versus metachronous disease (Table 3), tPSMA-TV% remained associated with lower odds of EBP (OR 0.66, 95% CI 0.52–0.84; *p* < 0.001) and higher odds of PSA50 response (OR 1.34, 95% CI 1.08–1.65; *p* = 0.007; q = 0.001 and q = 0.007, respectively). Neither log-transformed baseline PSA nor de novo versus metachronous disease reached statistical significance in either model.

### 3.2. Secondary Endpoints

In univariable Cox regression, higher baseline tPSMA-TV% was significantly associated with a lower risk of composite PSA-PFS event. For every 10% increase in tPSMA-TV%, the hazard of PSA-PFS event occurrence decreased by approximately 25% (HR 0.75, 95% CI 0.67–0.84; *p* < 0.001). Similarly, higher baseline tPSMA-TV% was significantly associated with improved OS. For every 10% increase in tPSMA-TV%, the hazard of death decreased by approximately 19% (HR 0.81, 95% CI 0.72–0.91; *p* < 0.001).

In multivariable Cox regression adjusted for log-transformed baseline PSA and de novo versus metachronous disease (Table 3), baseline tPSMA-TV% remained associated with PSA-PFS (HR 0.75, 95% CI 0.66–0.84; *p* < 0.001). In the multivariable OS model, tPSMA-TV% also remained associated with OS (HR 0.82, 95% CI 0.73–0.92; *p* < 0.001). In this model, log-transformed baseline PSA was also significantly associated with OS (HR 1.17, 95% CI 1.02–1.34; *p* = 0.021), whereas de novo versus metachronous disease was not.

### 3.3. Exploratory Endpoints

**Figure 2:**
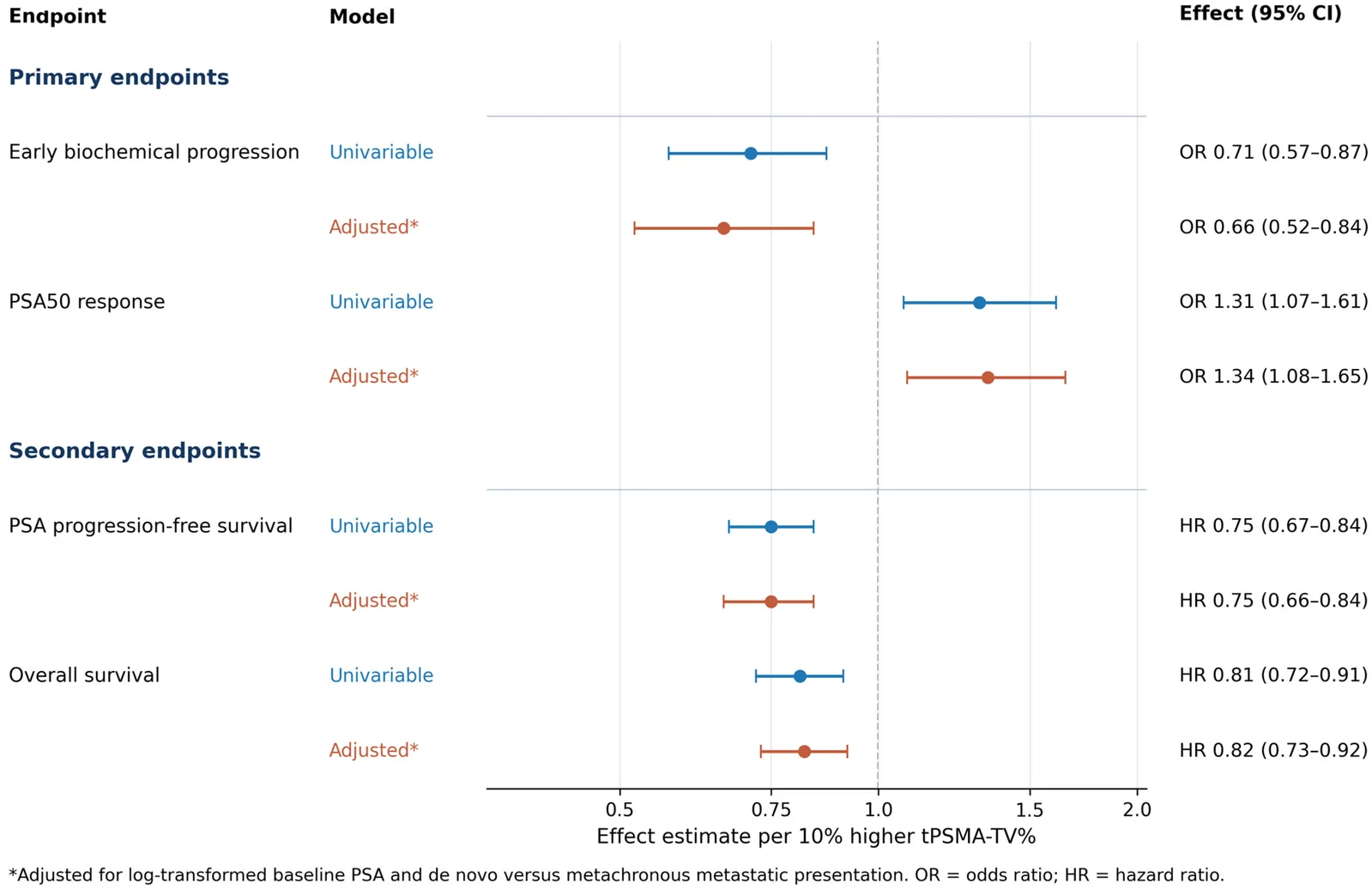
Univariable and multivariable associations between baseline tPSMA-TV% and the primary and secondary endpoints. Effect estimates are shown per 10% increase in tPSMA-TV% on a log scale.

Among the remaining PSMA PET-derived variables, several were associated with PSA-PFS in univariable Cox models, including SUVmean, RL-SUVmax, tPSMA-TV, and rTLQ%. For OS, significant univariable associations were observed for SUVmean, TLQ, tPSMA-TV, and rTLQ%. For EBP, significant univariable associations were observed for SUVmean, tPSMA-TV20, and rTLQ%, in addition to tPSMA-TV% (Figure 2).

**Figure 3:**
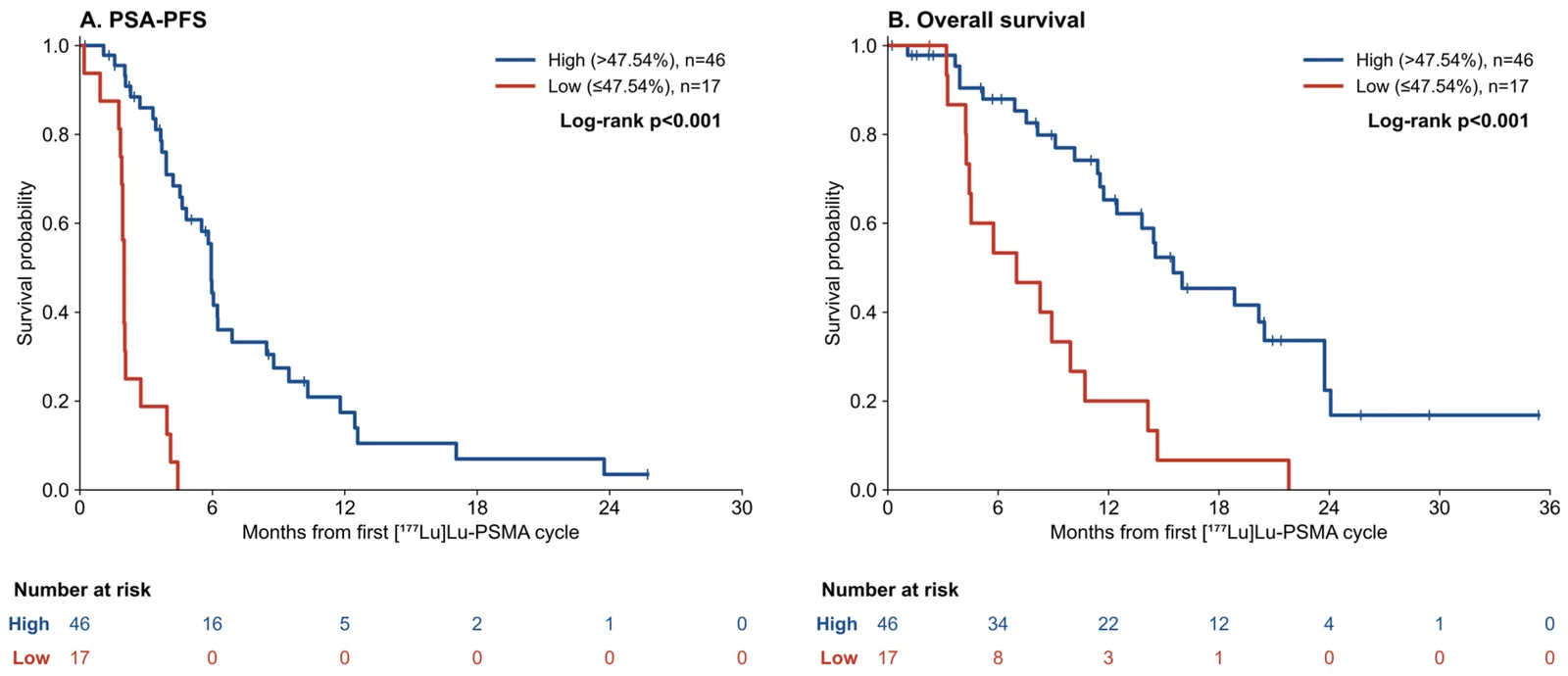
Kaplan–Meier estimates of PSA-PFS (**A**) and OS (**B**) according to the exploratory baseline tPSMA-TV% cut-off (low, ≤47.54%; high, >47.54%). Curves compare patients with high versus low high-uptake PSMA-positive tumor volume fraction.

ROC analysis identified an exploratory cut-off of 47.54% for tPSMA-TV% to discriminate EBP, with an AUC of 0.73 (95% CI, 0.57–0.86), sensitivity of 0.61 (14/23; 95% CI, 0.41–0.78) and specificity of 0.93 (37/40; 95% CI, 0.80–0.97). Using this cut-off (low tPSMA-TV%, ≤47.54%; n = 17), patients with low tPSMA-TV% had shorter PSA-PFS (HR 8.43, 95% CI 3.92–18.13; *p* < 0.001) and OS (HR 3.57, 95% CI 1.82–7.01; *p* < 0.001). These analyses are exploratory because the cut-off was derived and tested within the same cohort; hazard ratios obtained after data-driven dichotomisation are inflated and should not be interpreted as validated effect sizes, and the continuous analysis remains the primary result (Figure 3).

In exploratory logistic regression analysis, higher tPSMA-TV% was associated with a greater likelihood of PSA50 response (OR 1.31 per 10% increase, 95% CI 1.07–1.61; *p* = 0.009). Using the exploratory cut-off, low versus high tPSMA-TV% was associated with substantially lower odds of PSA50 response (OR 0.15, 95% CI 0.04–0.60; *p* = 0.007).

There was no statistically significant association between tPSMA-TV% and the number of completed [^177^Lu]Lu-PSMA cycles (Spearman rho = 0.20, *p* = 0.110). Age and liver involvement were not significantly associated with PSA-PFS or OS in univariable exploratory analyses.

A sensitivity analysis restricted to patients who completed at least two cycles yielded directionally consistent results for PSA-PFS, OS, and EBP. The detailed results of the exploratory analyses are set out in the tables and images contained in the Supplementary Materials.

### 3.4. Relationship with Established PET Parameters, Technical Heterogeneity and EBP Ascertainment

tPSMA-TV% correlated strongly with rTLQ% (Spearman ρ = 0.89) and moderately with SUVmean (ρ = 0.69), RL-SUVmax (ρ = 0.63), TLQ20 (ρ = 0.63) and tPSMA-TV20 (ρ = 0.56), whereas it was nearly uncorrelated with tPSMA-TV (ρ = −0.11) and TLQ (ρ = 0.20) (Supplementary Table S6). tPSMA-TV% therefore describes the distribution of uptake largely independently of tumor burden, but overlaps with average uptake.

When tPSMA-TV% was added to a model including log2-transformed tPSMA-TV and SUVmean, it remained associated with PSA-PFS (HR per 10% increase, 0.81; 95% CI, 0.71–0.92; likelihood ratio *p* = 0.001) and OS (HR, 0.82; 95% CI, 0.72–0.95; *p* = 0.006), whereas its associations with EBP (OR, 0.79; 95% CI, 0.61–1.04; *p* = 0.09) and PSA50 response (OR, 1.18; 95% CI, 0.92–1.51; *p* = 0.19) were attenuated and no longer significant. Gains in discrimination were small and imprecise for all endpoints: the C-index increased by 0.013 (95% CI, −0.023 to 0.060) for PSA-PFS and 0.023 (−0.017 to 0.095) for OS, and the AUC by 0.014 (−0.020 to 0.078) for EBP and 0.021 (−0.014 to 0.093) for PSA50 response (Supplementary Table S7).

tPSMA-TV% did not differ between patients imaged with [^68^Ga]Ga-PSMA-11 and [^18^F]F-PSMA-1007 (median 62.5% vs. 57.5%; *p* = 0.59). After adjustment for tracer, the estimates were materially unchanged (EBP OR 0.67, 95% CI 0.53–0.85; PSA50 OR 1.43, 1.12–1.82; PSA-PFS HR 0.72, 0.63–0.82; OS HR 0.81, 0.72–0.92), and there was no evidence of effect modification by tracer (interaction *p* ≥ 0.08). tPSMA-TV% was lower in patients treated with [^177^Lu]Lu-PSMA-617 than with [^177^Lu]Lu-PSMA-I&T (median 55.4% vs. 65.4%; *p* = 0.010), who also had a larger tPSMA-TV; adjustment for radioligand did not change the estimates, and interaction *p* values were ≥0.15 (Supplementary Table S8). Subgroup estimates were directionally consistent but imprecise.

Excluding the seven patients whose EBP status could not be ascertained, the association between tPSMA-TV% and EBP was unchanged (OR 0.69, 95% CI 0.54–0.87; adjusted OR 0.64, 0.49–0.83). When the three early deaths were also counted as events, the association was attenuated but remained significant (OR 0.76, 95% CI 0.61–0.93; *p* = 0.010) (Supplementary Table S9).

## 4. Discussion

In this retrospective cohort of mCRPC patients treated with [^177^Lu]Lu-PSMA-RLT, baseline tPSMA-TV% was consistently associated with lower odds of EBP and higher odds of PSA50 response, as well as longer PSA-PFS and OS. These associations remained significant after adjustment for baseline PSA and metastatic presentation (de novo versus metachronous), suggesting that tPSMA-TV% may provide prognostic information beyond selected clinical variables. Unlike conventional volumetric parameters such as tPSMA-TV, which mainly reflect disease extent, or uptake metrics such as SUVmean, which describe average target expression, tPSMA-TV% captures the relative fraction of tumor burden with intense PSMA expression above a predefined threshold.

This may be clinically relevant because highly PSMA-avid tumor deposits are more likely to be effectively targeted by PSMA-directed radioligand therapy, whereas a low tPSMA-TV% may indicate a larger proportion of weakly PSMA-expressing disease, potentially reflecting tumor heterogeneity, partial dedifferentiation, or less favourable target expression. This is consistent with previous observations that patients with higher PSMA uptake derive greater benefit from [^177^Lu]Lu-PSMA-RLT, whereas low-uptake lesions may represent biologically aggressive or less targetable disease [5,11,14]. The association with EBP is particularly relevant, as early PSA progression after [^177^Lu]Lu-PSMA therapy has been associated with poor clinical outcome and may indicate limited benefit from continued treatment [6]. The proportion of highly PSMA-avid tumor volume may capture a biologically meaningful aspect of disease heterogeneity relevant to treatment response.

In our cohort, each 10% increase in tPSMA-TV% was associated with a 29% reduction in the odds of EBP, a 31% increase in the odds of PSA50 response, a 25% reduction in the hazard of composite PSA-PFS event, and a 19% reduction in the hazard of death. These associations remained significant for both primary endpoints in univariable and multivariable models. The exploratory cut-off analysis was directionally consistent, identifying a subgroup with markedly shorter PSA-PFS and OS and lower likelihood of PSA50 response; however, because the cut-off was derived and tested within the same dataset, it should be considered hypothesis-generating only, requiring external validation. The cut-off favoured specificity over sensitivity (0.93 versus 0.61), making it better suited to identifying a high-confidence, poor-prognosis subgroup than to ruling out EBP risk, and is useful mainly as a visualization and clinical translation tool.

Our findings are in line with previous studies showing that semiquantitative PSMA-PET parameters are associated with outcome after [^177^Lu]Lu-PSMA-RLT. In the TheraP biomarker analysis, higher PSMA uptake was associated with greater benefit from [^177^Lu]Lu-PSMA-617 compared with cabazitaxel [27]. The VISION imaging substudy showed longer survival among patients with higher baseline PSMA uptake [28], and data from an expanded-access programme confirmed the prognostic value of total tumor SUVmean for PSA-PFS, OS, and PSA50 response [29]. A recent meta-analysis further supported the prognostic role of SUVmean and tumor volume, highlighting the complementary contribution of uptake intensity and disease burden metrics [30]. In this context, tPSMA-TV% does not replace established parameters but may add a further layer of information by describing how much of the total tumor volume demonstrates high target expression. In our cohort it correlated moderately with SUVmean and strongly with rTLQ%, and after adjustment for tPSMA-TV and SUVmean it remained associated with PSA-PFS and OS but not with the binary response endpoints, with small and imprecise gains in discrimination. tPSMA-TV% should therefore be regarded as a complementary descriptor of uptake heterogeneity; its incremental value over established parameters is not demonstrated in this cohort and requires formal model comparison in larger datasets.

The relationship between tumor burden and PSMA uptake heterogeneity is also relevant. In our cohort, tPSMA-TV was associated with PSA-PFS and OS, and TLQ with OS, confirming that disease burden remains clinically relevant, consistent with previous studies showing PSMA PET-derived total tumor volume and total lesion PSMA are associated with survival after [^177^Lu]Lu-PSMA-RLT [15,31]. The near absence of correlation between tPSMA-TV% and tPSMA-TV in our data supports this distinction. Tumor burden and uptake heterogeneity should therefore be viewed as complementary rather than competing biomarkers: patients with greater total tumor burden generally have worse outcomes, but among those with PSMA-positive disease, a higher relative proportion of intensely PSMA-avid volume may indicate a more targetable phenotype.

The prognostic hierarchy of PSMA PET-derived biomarkers may also vary by treatment setting. In the ENZA-p substudy, baseline PSMA-TTV retained prognostic and predictive value when [^177^Lu]Lu-PSMA-617 was combined with enzalutamide, whereas SUVmean was less informative [32]. Interim imaging may add further information: increased PSMA-TTV at 3 months in ENZA-p was associated with worse OS regardless of treatment arm and PSA response [33]. Changes in volumetric parameters after two cycles have been reported to predict OS and may outperform PSA kinetics [34], and post-treatment scintigraphic uptake has been associated with PSA response and progression risk [35]. Together, these data support broader integration of semiquantitative PSMA imaging into patient selection, response assessment, and trial design.

The clinical appeal of tPSMA-TV% lies in its simplicity: unlike complex radiomic or artificial intelligence-derived models, it can be obtained from standard semi-automated segmentation and expressed as an intuitive percentage. If confirmed in larger cohorts, it could contribute to baseline risk stratification before [^177^Lu]Lu-PSMA-RLT, helping identify patients at higher risk of early failure who may require closer monitoring. Whether such patients would benefit more from alternative or combined strategies is a hypothesis that requires testing in comparative cohorts or randomised trials, for which tPSMA-TV% could serve as a stratification factor [16,36,37].

Several biological mechanisms may underlie these associations. The absorbed tumor dose delivered by [^177^Lu]Lu-PSMA-617 correlates with tumor uptake on pretherapeutic PSMA-PET, and patients receiving a whole-body tumor dose below 10 Gy rarely achieve a PSA50 response [38]. A higher fraction of intensely avid volume is therefore expected to translate into a higher dose to a larger part of the disease. Conversely, a low tPSMA-TV% may reflect lineage plasticity with loss of androgen receptor signalling and suppression of PSMA expression, as described in neuroendocrine differentiation [39], or poorly perfused and hypoxic regions with lower ligand delivery and reduced radiosensitivity. Because tPSMA-TV% is computed only within PSMA-positive volumes, it cannot capture PSMA-negative, [^18^F]FDG-avid disease, which is associated with poor outcome after [^177^Lu]Lu-PSMA-617 [27]; our data cannot distinguish between these explanations.

Several limitations should be acknowledged. The study was retrospective and single-centre, with a modest sample size limiting extensive multivariable modelling, and residual confounding cannot be excluded. The cohort included patients imaged with either [^68^Ga]Ga-PSMA-11 or [^18^F]F-PSMA-1007 and treated with different PSMA radioligands, reflecting real-world practice but introducing technical and biological heterogeneity [40]. Adjusted and stratified analyses did not suggest that tracer or radioligand modified the association, but subgroups were small. Because each patient was imaged with a single tracer, the equivalence of the SUV 20 threshold between [^68^Ga]Ga-PSMA-11 and [^18^F]F-PSMA-1007 could not be assessed; this would require intra-individual comparison of both tracers. The SUV threshold of 20, although clinically intuitive, is not universally standardised and may be influenced by scanner calibration, reconstruction protocols, tracer characteristics, and uptake time [25]; both the reproducibility of tPSMA-TV% and the optimal threshold therefore require prospective validation. Inter-observer agreement was not formally assessed, and alternative thresholds (for example, SUV 15 or 25) could not be evaluated because segmentation was performed only at SUV 20. The absence of [^18^F]FDG PET/CT, lesion-level dosimetry, and molecular or genomic characterisation is a further limitation, as these could help distinguish low PSMA uptake due to dedifferentiated or biologically aggressive disease from other causes of reduced tracer accumulation [41]. Early biochemical progression was also defined operationally from available follow-up data rather than as a prospectively recorded endpoint, and EBP could not be ascertained in seven patients with less than 4 months of PSA follow-up, although excluding them did not change the results. The exploratory cut-off should not be regarded as a validated decision limit, and external validation in an independent multicentre cohort is required.

## 5. Conclusions

In this real-world study, baseline tPSMA-TV% was associated with lower odds of EBP and higher odds of PSA50 response, and secondarily with PSA-PFS and OS, in patients with mCRPC undergoing [^177^Lu]Lu-PSMA-RLT, after adjustment for baseline PSA and metastatic presentation. These findings support the relevance of high-uptake tumor volume fraction as a simple, interpretable candidate prognostic imaging biomarker that may complement established measures of tumor burden and global PSMA uptake. Its incremental value over established PSMA-PET parameters and its reproducibility across tracers and readers remain to be established, and prospective multicentre validation is warranted before routine clinical application.

## Figures and Tables

**Table 1:** Baseline characteristics of patients.

Characteristic	Data
Patients (n)	63
Age at baseline, years	81 (72–84)
De novo metastatic disease	32 (50.8%)
Metachronous metastatic disease	31 (49.2%)
Treatment history	
Previous docetaxel	35 (55.6%)
Second-line chemotherapy	15 (23.8%)
Androgen receptor signaling inhibitor	63 (100%)
Ra223	16 (25.4%)
Baseline PSA, ng/mL	42.6 (8.4–216.5)
ALP, U/L	91 (67–157)
Liver involvement	14 (22.2%)
Days between baseline PSMA PET/CT and [^177^Lu]Lu-PSMA-RLT	41 (28–56)
Cycles completed	3 (2–4)
[^177^Lu]Lu-PSMA-617	36 (57.1%)
[^177^Lu]Lu-PSMA-I&T	27 (42.9%)
[^68^Ga]Ga-PSMA-11	33 (52.4%)
[^18^F]F-PSMA-1007	30 (47.6%)
Baseline PSMA PET parameters	
RL-SUVmax	52.4 (35.5–75.3)
SUVmean	17.0 (12.9–23.4)
TLQ, SUV × mL	1343.9 (547.5–2687.5)
TLQ20, SUV × mL	563.3 (149.4–2174.5)
tPSMA-TV, mL	88.3 (28.2–167.4)
tPSMA-TV20, mL	26.3 (8.6–94.2)
tPSMA-TV%, %	61.4 (46.2–73.8)
rTLQ%, %	75.8 (42.2–90.1)
Outcome	
Composite PSA-PFS event	51 (81.0%)
Death	40 (63.5%)
EBP	23 (36.5%)
PSA50 response	30 (47.6%)
Median PSA-PFS, months (95% CI)	4.4 (3.7–6.0)
Median OS, months (95% CI)	13.8 (9.9–15.5)
Median follow-up, months (95% CI)	20.9 (15.4–29.4)

Values are median (IQR) or n (%), except for PSA-PFS, OS and follow-up, which are Kaplan–Meier medians (95% CI); follow-up was estimated with the reverse Kaplan–Meier method.

**Table 2:** Univariable analyses of primary endpoints.

Endpoint	Predictor	Effect (95% CI)	*p*	N	Events
EBP	tPSMA-TV% per +10%	OR 0.71 (0.57–0.87)	0.001	63	23
PSA50 response	tPSMA-TV% per +10%	OR 1.31 (1.07–1.61)	0.009	63	30

**Table 3:** Multivariable analyses of primary and secondary endpoints.

Endpoint	Predictor	Effect (95% CI)	*p*
EBP	tPSMA-TV% per +10%	OR 0.66 (0.52–0.84)	<0.001
EBP	log(Baseline PSA + 1)	OR 0.90 (0.69–1.18)	0.455
EBP	de novo vs. metachronous	OR 0.28 (0.07–1.08)	0.064
PSA50 response	tPSMA-TV% per +10%	OR 1.34 (1.08–1.65)	0.007
PSA50 response	log(Baseline PSA + 1)	OR 1.23 (0.94–1.59)	0.131
PSA50 response	de novo vs. metachronous	OR 1.14 (0.37–3.48)	0.818
PSA-PFS	tPSMA-TV% per +10%	HR 0.75 (0.66–0.84)	<0.001
PSA-PFS	log(Baseline PSA + 1)	HR 1.01 (0.90–1.14)	0.835
PSA-PFS	de novo vs. metachronous	HR 0.69 (0.38–1.26)	0.233
OS	tPSMA-TV% per +10%	HR 0.82 (0.73–0.92)	<0.001
OS	log(Baseline PSA + 1)	HR 1.17 (1.02–1.34)	0.021
OS	de novo vs. metachronous	HR 1.16 (0.62–2.20)	0.639

## Data Availability

The data supporting the reported results are available upon request.

## Supplementary Materials

The following supporting information can be downloaded at https://www.mdpi.com/article/10.3390/cancers18203297/s1. Supplementary Methods S1; Supplementary Results S1; Table S1: Exploratory univariable models for PSA-PFS; Table S2: Exploratory univariable models for OS; Table S3: Exploratory univariable models for EBP; Table S4: Additional exploratory analyses; Table S5: Supplementary PSA progression-only models with deaths censored; Figure S1: tPSMA-TV% by EBP status; Figure S2: ROC curve for EBP; Figure S3: Other PSMA PET metrics for PSA-PFS, OS, and EBP; Figure S4: Sensitivity analysis in patients with >=2 cycles; Figure S5: PSA progression-only endpoint with deaths before documented PSA progression censored; Figure S6: Patient flow diagram; Table S6: Spearman correlations between PSMA-PET parameters; Table S7: Incremental value of tPSMA-TV% over tPSMA-TV and SUVmean; Table S8: Analyses by PET tracer and therapeutic radioligand; Table S9: Sensitivity analyses of EBP ascertainment.
