# Supplementary material for: Prognostic Value of Baseline High-Uptake PSMA Tumor Volume Fraction in Metastatic Castration-Resistant Prostate Cancer Patients Treated with [177Lu]Lu-PSMA Radioligand Therapy
Source: Cancers (Basel). 2026 Oct 13;18(20):3297. doi: 10.3390/cancers18203297 (PMC13702756; doi:10.3390/cancers18203297)
Supplement: Supplementary file 1 [file cancers-18-03297-s001.zip › cancers-4595987-supplementary.pdf]

Supplementary Materials

Contents

Supplementary Methods S1
Supplementary Results S1. Exploratory analyses: other baseline PET-derived variables
Table S1. Exploratory univariable models for PSA-PFS
Table S2. Exploratory univariable models for OS
Table S3. Exploratory univariable models for EBP
Table S4. Additional exploratory analyses
Table S5. Supplementary PSA progression-only models with deaths censored
Figure S1. tPSMA-TV% by EBP status
Figure S2. ROC curve for EBP
Figure S3. Other PSMA PET metrics for PSA-PFS, OS, and EBP
Figure S4. Sensitivity analysis in patients with >=2 cycles
Figure S5. PSA progression-only endpoint with deaths before documented PSA progression censored
Figure S6. Patient flow diagram
Table S6. Spearman correlations between PSMA-PET parameters
Table S7. Incremental value of tPSMA-TV% over tPSMA-TV and SUVmean
Table S8. Analyses by PET tracer and therapeutic radioligand
Table S9. Sensitivity analyses of EBP ascertainment

Supplementary Methods S1

The methodological definitions are consistent with the main manuscript. Additional details relevant to image segmentation, exploratory analyses, and supplementary sensitivity analyses are reported below.

Study Design

This retrospective, single-centre, observational study included patients with mCRPC referred to our institution for [^{177}Lu]Lu-PSMA-RLT. Eligibility criteria were: (1) availability of baseline PSMA PET/CT ([^{68}Ga]Ga-PSMA-11 or [^{18}F]F-PSMA-1007), (2) completion of at least one cycle of [^{177}Lu]Lu-PSMA-RLT ([^{177}Lu]Lu-PSMA-617 or [^{177}Lu]Lu-PSMA-I&T), and (3) sufficient follow-up data for EBP, PSA50 response, PSA-PFS, and survival analyses.

For each patient, we collected age, date of initial diagnosis, ISUP grade, disease presentation (de novo versus metachronous metastatic disease), serum PSA values at initial diagnosis and during [^{177}Lu]Lu-PSMA-RLT, treatments performed before [^{177}Lu]Lu-PSMA-RLT, pre-treatment alkaline phosphatase and haemoglobin values, liver involvement, number of lesions, PSMA PET/CT quantitative parameters, biochemical failure, and survival outcomes.

The study was conducted within a retrospective data-collection protocol approved by the local ethics committee (protocol number 2023-01208) and in accordance with the Declaration of Helsinki and later amendments or comparable ethical standards.

Radioligand Therapy Protocol

All patients received intravenous [^{177}Lu]Lu-PSMA-RLT at 6- to 8-week intervals, following EANM guidelines. The administered activity per cycle was 7.4 GBq (+/-5%). Corticosteroids, proton pump inhibitors, metoclopramide, and hydration were administered according to institutional protocol to mitigate side effects.

PSMA PET/CT Imaging Acquisition and Segmentation

Whole-body imaging was performed 60 +/- 5 minutes after intravenous injection of either [^{18}F]F-PSMA-1007 or [^{68}Ga]Ga-PSMA-11 at a mean administered activity of 3.7 MBq/kg. PET/CT scans were obtained on a Biograph mCT40 system (Siemens Medical Solutions, Germany), applying standard protocols in accordance with European Association of Nuclear Medicine guidelines. PET/CT was acquired with continuous flowing bed motion using the same axial field as the CT scan, and PET images were reconstructed with standard iterative 3D algorithms including time-of-flight and point-spread-function corrections.

PET/CT images were interpreted and analysed by two expert nuclear medicine physicians (MC and GPa), blinded to patients' clinical and pathological information. Quantitative SUV-derived parameters were obtained using dedicated software (MM Oncology, Lesion Scout, SyngoVia v.8.0, Siemens Medical Solutions, Germany). For each patient, the volume of interest (VOI) encompassing all PSMA avid tumor lesions was derived by semi-automatic segmentation using a threshold of 40% of SUVmax. Organs with physiological PSMA uptake were excluded from the analysis, and volumes adjacent to physiologically avid organs or urinary activity were corrected manually. tPSMA-TV20 was obtained by applying a fixed threshold of SUV 20 to the voxels within the tumor VOIs, and is therefore a voxel-wise sub-volume of tPSMA-TV rather than the volume of lesions with SUVmax ≥ 20. TLQ is the product of SUVmean and tPSMA-TV (analogous to total lesion PSMA), TLQ20 the corresponding product within tPSMA-TV20, and rTLQ% = 100 × TLQ20 / TLQ. The reference lesion was the lesion with the highest SUVmax. Readings were performed in consensus; inter-observer agreement was not formally assessed.

Clinical Endpoints and Definitions

The co-primary endpoints were early biochemical progression (EBP) and PSA50 response; PSA-PFS and OS were secondary endpoints. Because no standardized terminology currently exists to describe PSA progression occurring very early after treatment initiation, we defined this outcome as Early Biochemical Progression (EBP)—PCWG3-defined PSA progression within 4 months of [^{177}Lu]Lu-PSMA-RLT initiation—to capture patients who derive minimal or no benefit from treatment and in whom early identification could most directly inform treatment decisions. PSA50 response was defined as a PSA decline of at least 50% from baseline.

Death alone before documented PSA progression was not counted as EBP. EBP was ascertained from the PSA values measured before each cycle and during follow-up; patients alive without documented PSA progression were classified as not having EBP. Three patients died within 4 months without documented PSA progression, and 7 patients were alive without PSA progression after less than 4 months of PSA follow-up; their EBP status is examined in the sensitivity analyses of Table S9. PSA-PFS, a secondary endpoint, was defined as the time from the first Lu-PSMA cycle to PCWG3-defined PSA progression or death from any cause, whichever occurred first. Patients alive without PSA progression were censored at last available follow-up. A PSA progression-only endpoint, in which deaths before documented PSA progression were censored, was evaluated as a supplementary sensitivity analysis. OS, a secondary endpoint, was defined as time from the first Lu-PSMA cycle to death from any cause or last follow-up.

Multivariable analyses assessed whether the association between baseline tPSMA-TV% and each primary and secondary endpoint remained significant after adjustment for log-transformed baseline PSA and de novo versus metachronous metastatic presentation.

Exploratory analyses included: (1) univariable assessment of other PSMA PET-derived variables for PSA-PFS, OS, and EBP; (2) identification of the ROC-derived tPSMA-TV% cut-off for EBP; (3) Cox models for PSA-PFS and OS using tPSMA-TV% as a

categorical variable based on the exploratory cut-off; (4) categorical cut-off analyses for PSA50 response; (5) exploratory association of tPSMA-TV% with completed Lu-PSMA cycles; (6) evaluation of age; (7) evaluation of liver involvement; and (8) supplementary PSA progression-only models in which deaths before documented PSA progression were censored.

Statistical Analysis

Continuous variables were summarised as median and interquartile range (IQR), and categorical variables as number and percentage. The main biomarker, tPSMA-TV%, was analysed as a continuous variable in all primary and secondary endpoint analyses. Cox proportional hazards regression was used for the secondary endpoints PSA-PFS and OS, and logistic regression for the primary endpoints EBP and PSA50 response. Effect estimates are reported as hazard ratios (HRs) for Cox models and odds ratios (ORs) for logistic models, with 95% confidence intervals (95% CI). Cox models used Breslow handling of ties.

To improve interpretability, tPSMA-TV% and rTLQ% were modelled per 10% increase, RL-SUVmax per 10-unit increase, SUVmean per 5-unit increase, and right-skewed volumetric variables (TLQ, TLQ20, tPSMA-TV, and tPSMA-TV20) per doubling after log2 transformation.

For exploratory ROC analysis, the optimal tPSMA-TV% cut-off for EBP was identified using the Youden index. Because this cut-off was derived and tested within the same dataset, it was considered hypothesis-generating only. Patients with tPSMA-TV% ≤ 47.54% were classified as low. 95% confidence intervals were obtained by bootstrap (1000 resamples) for the AUC and with the Wilson method for sensitivity, specificity and predictive values. Median follow-up was estimated with the reverse Kaplan–Meier method. Incremental value was assessed by adding tPSMA-TV% (per 10%) to a model including log2(tPSMA-TV + 1) and SUVmean (per 5 units), using the likelihood ratio test and bootstrap 95% confidence intervals for the change in AUC or Harrell's C-index. Tracer and radioligand were entered as covariates and as interaction terms with tPSMA-TV%, and models were also fitted within each subgroup. Exploratory analyses across multiple PET variables were additionally summarised using false discovery rate (FDR)-adjusted q-values within each endpoint family. All tests were two-sided, and p values <0.05 were considered statistically significant. Analyses were performed using Python 3.12.13 with pandas 3.0.1 and NumPy 2.3.5.

Supplementary Results S1. Exploratory Analyses: Other Baseline PET-Derived Variables

This section contains the exploratory PET-derived variable analyses. Tables S1 and S2 report univariable models for the secondary endpoints PSA-PFS and OS, respectively; Table S3 reports exploratory associations with the primary endpoint EBP. Tables S4 and S5 summarise additional exploratory analyses, including the ROC-derived tPSMA-TV% cut-off, categorical cut-off models and Cox regression models for PSA progression only.

Table S1. Exploratory univariable models for PSA-PFS.

Predictor	Effect (95% CI)	p	q
tPSMA-TV% (+10%)	HR 0.75 (0.67-0.84)	<0.001	<0.001
RL-SUVmax (+10)	HR 0.89 (0.81-0.97)	0.008	0.013
SUVmean (+5)	HR 0.70 (0.57-0.86)	<0.001	0.002
TLQ (per doubling)	HR 1.11 (0.94-1.31)	0.225	0.257
TLQ20 (per doubling)	HR 0.91 (0.82-1.02)	0.108	0.144
tPSMA-TV (per doubling)	HR 1.24 (1.07-1.45)	0.005	0.010

Predictor	Effect (95% CI)	p	q
tPSMA-TV20 (per doubling)	HR 0.97 (0.85-1.11)	0.674	0.674
rTLQ% (+10%)	HR 0.87 (0.80-0.94)	<0.001	0.002

Table S2. Exploratory univariable models for OS.

Predictor	Effect (95% CI)	p	q
tPSMA-TV% (+10%)	HR 0.81 (0.72-0.91)	<0.001	0.001
RL-SUVmax (+10)	HR 0.93 (0.85-1.01)	0.098	0.131
SUVmean (+5)	HR 0.78 (0.64-0.95)	0.012	0.023
TLQ (per doubling)	HR 1.47 (1.18-1.84)	<0.001	0.001
TLQ20 (per doubling)	HR 1.01 (0.89-1.14)	0.923	0.923
tPSMA-TV (per doubling)	HR 1.65 (1.33-2.04)	<0.001	<0.001
tPSMA-TV20 (per doubling)	HR 1.09 (0.92-1.29)	0.320	0.366
rTLQ% (+10%)	HR 0.90 (0.83-0.98)	0.015	0.023

Table S3. Exploratory univariable models for EBP.

Predictor	Effect (95% CI)	p	q
tPSMA-TV% (+10%)	OR 0.71 (0.57-0.87)	0.001	0.010
RL-SUVmax (+10)	OR 0.91 (0.79-1.05)	0.188	0.251
SUVmean (+5)	OR 0.50 (0.31-0.81)	0.005	0.019
TLQ (per doubling)	OR 0.94 (0.68-1.31)	0.728	0.728
TLQ20 (per doubling)	OR 0.82 (0.69-0.99)	0.036	0.058
tPSMA-TV (per doubling)	OR 1.21 (0.88-1.67)	0.245	0.280
tPSMA-TV20 (per doubling)	OR 0.74 (0.57-0.95)	0.017	0.033
rTLQ% (+10%)	OR 0.83 (0.71-0.97)	0.017	0.033

Table S4. Additional exploratory analyses.

Analysis	Result
Best ROC cut-off for EBP (tPSMA-TV%)	≤47.54% (low)
AUC for EBP	0.73 (95% CI, 0.57–0.86)
Sensitivity	0.61 (14/23; 95% CI, 0.41–0.78)
Specificity	0.93 (37/40; 95% CI, 0.80–0.97)
Positive predictive value	0.82 (14/17; 95% CI, 0.59–0.94)
Negative predictive value	0.80 (37/46; 95% CI, 0.67–0.89)
PSA-PFS, low vs. high tPSMA-TV%	HR 8.43 (3.92–18.13), p<0.001
OS, low vs. high tPSMA-TV%	HR 3.57 (1.82–7.01), p<0.001
PSA50 response, low vs. high tPSMA-TV%	OR 0.15 (0.04–0.60), p=0.007
Spearman correlation between tPSMA-TV% and cycles completed	rho = 0.20, p=0.110
Age and PSA-PFS	HR 0.90 (0.65-1.26), p=0.548
Age and OS	HR 1.18 (0.79-1.77), p=0.427
Liver involvement and PSA-PFS	HR 1.11 (0.57-2.14), p=0.760
Liver involvement and OS	HR 1.30 (0.63-2.69), p=0.481

Table S5. Supplementary PSA progression-only models with deaths before documented PSA progression censored.

Endpoint	Model	Predictor	Effect (95% CI)	p	Events
PSA progression-only	Cox univariable	tPSMA-TV% per +10%	HR 0.73 (0.64-0.83)	<0.001	44
PSA progression-only	Cox multivariable	tPSMA-TV% per +10%	HR 0.72 (0.63-0.82)	<0.001	44
PSA progression-only	Cox multivariable	log(Baseline PSA + 1)	HR 0.97 (0.86-1.10)	0.675	44
PSA progression-only	Cox multivariable	De novo metastatic disease	HR 0.58 (0.30-1.11)	0.102	44

Tables S1, S2, and S3 summarise the univariable assessment of other PET-derived variables for PSA-PFS, OS, and EBP. PSMA PET-derived variables associated with composite PSA-PFS in univariable Cox models were tPSMA-TV%, RL-SUVmax, SUVmean, tPSMA-TV, and rTLQ%. For OS, significant univariable associations were observed for SUVmean, TLQ, tPSMA-TV, and rTLQ%. For EBP, significant univariable associations were observed for SUVmean, tPSMA-TV20, and rTLQ%, in addition to tPSMA-TV%.

ROC analysis identified an exploratory tPSMA-TV% cut-off of 47.54% for EBP, with an AUC of 0.73, sensitivity of 0.61, and specificity of 0.93. Using this cut-off, low tPSMA-TV% was associated with substantially worse PSA-PFS (HR 7.35, 95% CI 3.44-15.72; p<0.001) and OS (HR 3.52, 95% CI 1.78-6.94; p<0.001). These cut-off analyses should be interpreted as hypothesis-generating because the threshold was derived and tested in the same cohort.

Table S4 summarises the additional exploratory analyses of baseline tPSMA-TV% and outcomes after [^{177}Lu]Lu-PSMA-RLT. Results include the ROC-derived optimal cut-off for early biochemical progression (EBP), together with its discriminative performance (AUC, sensitivity, specificity); PSA-PFS, OS, and PSA50 response according to the low- versus high-tPSMA-TV% subgroups defined by this cut-off; the Spearman correlation between tPSMA-TV% and the number of treatment cycles completed; and univariable Cox analyses of age and liver involvement with PSA-PFS and OS. HR, hazard ratio; OR, odds ratio; AUC, area under the curve; CI, confidence interval.

Table S5 summarises the supplementary Cox regression models for PSA progression-free survival restricted to PSA progression events only, with deaths occurring before documented PSA progression censored at last follow-up. Univariable and multivariable associations between baseline tPSMA-TV% (per 10% increase) and PSA progression are shown, with the multivariable model additionally adjusted for baseline PSA and metastatic presentation (de novo versus metachronous). HR, hazard ratio; CI, confidence interval.

Table S6. Spearman correlation coefficients between baseline PSMA-PET parameters (n = 63).

Parameter	tPSMA-TV%	tPSMA-TV	tPSMA-TV20	SUVmean	RL-SUVmax	TLQ	TLQ20	rTLQ%
tPSMA-TV%	1.00	-0.11	0.56	0.69	0.63	0.20	0.63	0.89
tPSMA-TV	-0.11	1.00	0.59	-0.34	-0.18	0.89	0.46	-0.07
tPSMA-TV20	0.56	0.59	1.00	0.29	0.36	0.77	0.90	0.55
SUVmean	0.69	-0.34	0.29	1.00	0.75	0.08	0.47	0.70
RL-SUVmax	0.63	-0.18	0.36	0.75	1.00	0.14	0.52	0.74
TLQ	0.20	0.89	0.77	0.08	0.14	1.00	0.71	0.25
TLQ20	0.63	0.46	0.90	0.47	0.52	0.71	1.00	0.73
rTLQ%	0.89	-0.07	0.55	0.70	0.74	0.25	0.73	1.00

tPSMA-TV% is strongly correlated with rTLQ% and moderately with SUVmean, and nearly uncorrelated with tPSMA-TV and TLQ.

Table S7. Incremental value of tPSMA-TV% over log2(tPSMA-TV + 1) and SUVmean.

End-point	Discrimination metric	Base model	Base + tPSMA-TV%	Difference (95% CI)	LR test p	tPSMA-TV% effect in full model (95% CI)
EBP	AUC	0.754	0.768	+0.014 (-0.020 to +0.078)	0.092	OR 0.79 (0.61–1.04)
PSA50	AUC	0.745	0.767	+0.021 (-0.014 to +0.093)	0.185	OR 1.18 (0.92–1.51)
PSA-PFS	Harrell C	0.708	0.721	+0.013 (-0.023 to +0.060)	0.001	HR 0.81 (0.71–0.92)
OS	Harrell C	0.732	0.755	+0.023 (-0.017 to +0.095)	0.006	HR 0.82 (0.72–0.95)

Base model: log2(tPSMA-TV + 1) and SUVmean (per 5 units); full model adds tPSMA-TV% (per 10%). Logistic regression for EBP and PSA50 response (AUC), Cox regression for PSA-PFS and OS (Harrell's C-index). 95% CIs of the differences from 1000 bootstrap resamples; LR, likelihood ratio.

Table S8. Association of tPSMA-TV% (per 10% increase) with outcomes by PET tracer and therapeutic radioligand.

Factor	End-point	Adjusted for factor (95% CI)	p	Subgroup 1 (95% CI) [events]	Subgroup 2 (95% CI) [events]	Interaction p
Tracer	EBP	OR 0.67 (0.53–0.85)	<0.001	0.72 (0.53–0.99) [13]	0.63 (0.44–0.89) [10]	0.559
Tracer	PSA50	OR 1.43 (1.12–1.82)	0.004	1.14 (0.85–1.53) [12]	1.88 (1.17–3.02) [17]	0.080
Tracer	PSA-PFS	HR 0.72 (0.63–0.82)	<0.001	0.67 (0.53–0.85) [22]	0.72 (0.61–0.86) [28]	0.198
Tracer	OS	HR 0.81 (0.72–0.92)	<0.001	0.78 (0.62–0.96) [13]	0.83 (0.72–0.96) [26]	0.362
Radioligand	EBP	OR 0.69 (0.55–0.86)	0.001	0.63 (0.46–0.85) [14]	0.79 (0.57–1.10) [9]	0.308
Radioligand	PSA50	OR 1.40 (1.11–1.75)	0.004	1.25 (0.98–1.61) [18]	1.96 (1.11–3.46) [12]	0.154
Radioligand	PSA-PFS	HR 0.75 (0.66–0.84)	<0.001	0.69 (0.58–0.83) [29]	0.83 (0.69–0.99) [22]	0.487
Radioligand	OS	HR 0.82 (0.73–0.93)	0.002	0.79 (0.68–0.93) [25]	0.88 (0.72–1.08) [15]	0.400

Subgroup 1: [^{68}Ga]Ga-PSMA-11 (tracer) or [^{177}Lu]Lu-PSMA-617 (radioligand); subgroup 2: [^{18}F]F-PSMA-1007 or [^{177}Lu]Lu-PSMA-I&T. Tracer analyses in 62 patients (tracer not recorded in one). Tracer: [^{68}Ga]Ga-PSMA-11 (n = 33) median tPSMA-TV% 62.5% (IQR 50.8–76.8) vs. [^{18}F]F-PSMA-1007 (n = 29) 57.5% (IQR 44.5–70.7), Mann–Whitney p = 0.592; Radioligand: [177Lu]Lu-PSMA-617 (n = 36) median tPSMA-TV% 55.4% (IQR 15.1–68.1) vs. [^{177}Lu]Lu-PSMA-I&T (n = 27) 65.4% (IQR 55.8–81.8), Mann–Whitney p = 0.010. Unadjusted outcomes by radioligand ([^{177}Lu]Lu-PSMA-617 vs. [^{177}Lu]Lu-PSMA-I&T): PSA50 response 50% vs. 44%, EBP 39% vs. 33%, median PSA-PFS 4.5 vs. 4.2 months, median OS 10.2 vs. 15.5 months (log-rank p = 0.029); by tracer ([^{68}Ga]Ga-PSMA-11 vs. [^{18}F]F-PSMA-1007): PSA50 response 36% vs. 59%, EBP 39% vs. 34%, median PSA-PFS 3.7 vs. 5.5 months, median OS 11.7 vs. 14.4 months. These unadjusted comparisons are confounded by calendar period and by the larger tumor burden of patients treated with [^{177}Lu]Lu-PSMA-617, and do not compare the efficacy of the two radioligands.

Table S9. Sensitivity analyses of EBP ascertainment (tPSMA-TV% per 10% increase).

Analysis	n	EBP events	Univariable OR (95% CI)	p	Adjusted OR (95% CI)	p	AUC
Main analysis (all patients)	63	23	0.71 (0.57–0.87)	0.001	0.66 (0.52–0.84)	<0.001	0.73

Analysis	n	EBP events	Univariable OR (95% CI)	p	Adjusted OR (95% CI)	p	AUC
Excluding 7 patients with non-ascertainable EBP status	56	23	0.69 (0.54–0.87)	0.002	0.64 (0.49–0.83)	<0.001	0.74
Excluding non-ascertainable patients; 3 early deaths counted as events	56	26	0.76 (0.61–0.93)	0.010	0.74 (0.59–0.92)	0.008	0.67

Non-ascertainable: alive without documented PSA progression and less than 4 months of PSA follow-up. Adjusted models include log(baseline PSA + 1) and de novo versus metachronous presentation.

Abbreviations: AUC, area under the receiver-operating characteristic curve; CI, confidence interval; EBP, early biochemical progression; FDR, false discovery rate; HR, hazard ratio; OR, odds ratio; OS, overall survival; PET, positron emission tomography; PSA, prostate-specific antigen; PSA-PFS, prostate-specific antigen progression-free survival; PSMA, prostate-specific membrane antigen; ROC, receiver-operating characteristic; RL-SUVmax, maximum PSMA SUV expression of the reference lesion; SUV, standardized uptake value; TLQ, total lesion quotient; TLQ20, TLQ above SUV 20; tPSMA-TV, total PSMA tumor volume; tPSMA-TV20, tPSMA-TV above SUV 20; tPSMA-TV%, percentage of tPSMA-TV20 within tPSMA-TV.

Supplementary Figures

Figure S1. tPSMA-TV% by EBP Status

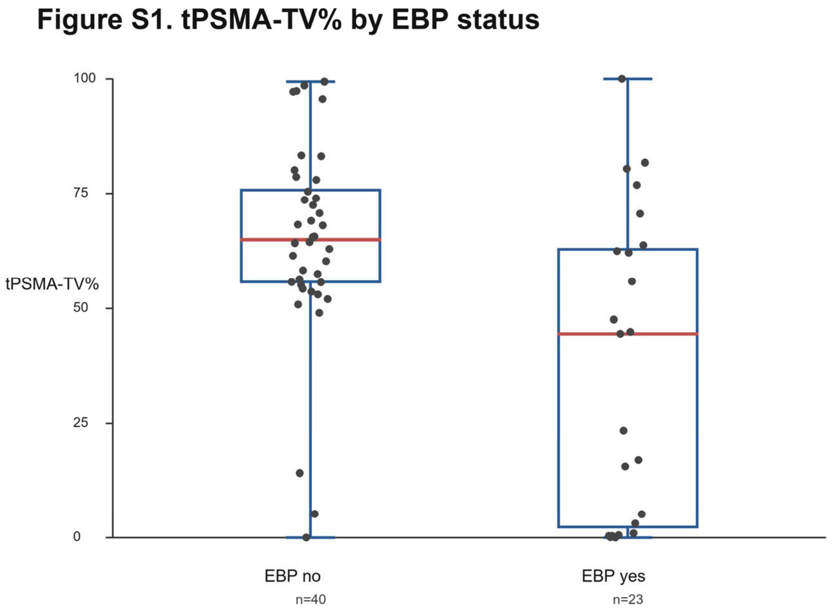

Figure S1. Distribution of baseline tPSMA-TV% according to EBP status. The boxplot compares patients with and without early biochemical progression.

Abbreviations: EBP, early biochemical progression; PET, positron emission tomography; PSMA, prostate-specific membrane antigen; tPSMA-TV%, percentage of high-uptake PSMA-positive tumor volume within total PSMA-positive tumor volume.

Figure S2. ROC Curve for EBP

Figure S2. ROC curve for EBP

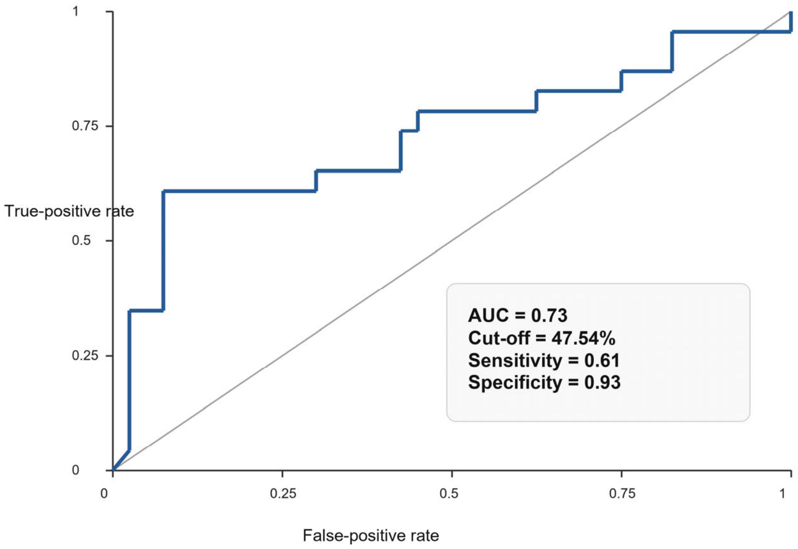

Figure S2. Receiver-operating characteristic curve for baseline tPSMA-TV% to discriminate EBP. The exploratory cut-off was 47.54%, with AUC 0.73, sensitivity 0.61, and specificity 0.93.

Abbreviations: AUC, area under the receiver-operating characteristic curve; EBP, early biochemical progression; ROC, receiver-operating characteristic; tPSMA-TV%, percentage of high-uptake PSMA-positive tumor volume within total PSMA-positive tumor volume.

Figure S3. Other PSMA PET Metrics for PSA-PFS, OS, and EBP

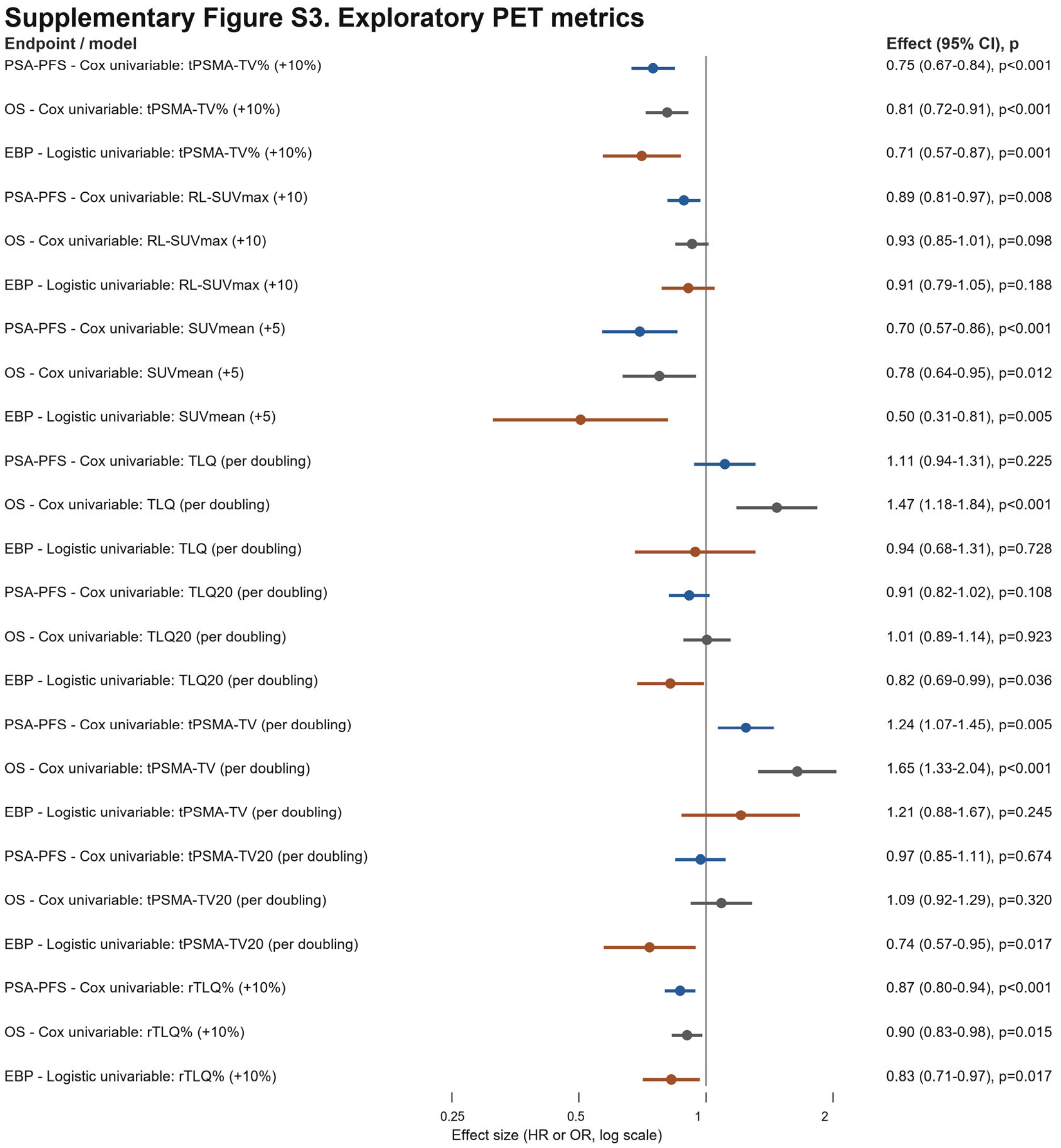

Figure S3. Exploratory univariable associations between baseline PSMA PET-derived metrics and PSA-PFS, OS, and EBP. Effect estimates are displayed on a log scale.

Abbreviations: CI, confidence interval; EBP, early biochemical progression; HR, hazard ratio; OR, odds ratio; OS, overall survival; PET, positron emission tomography; PSA-PFS, prostate-specific antigen progression-free survival; PSMA, prostate-specific membrane antigen.

Figure S4. Sensitivity Analysis in Patients with >=2 Cycles

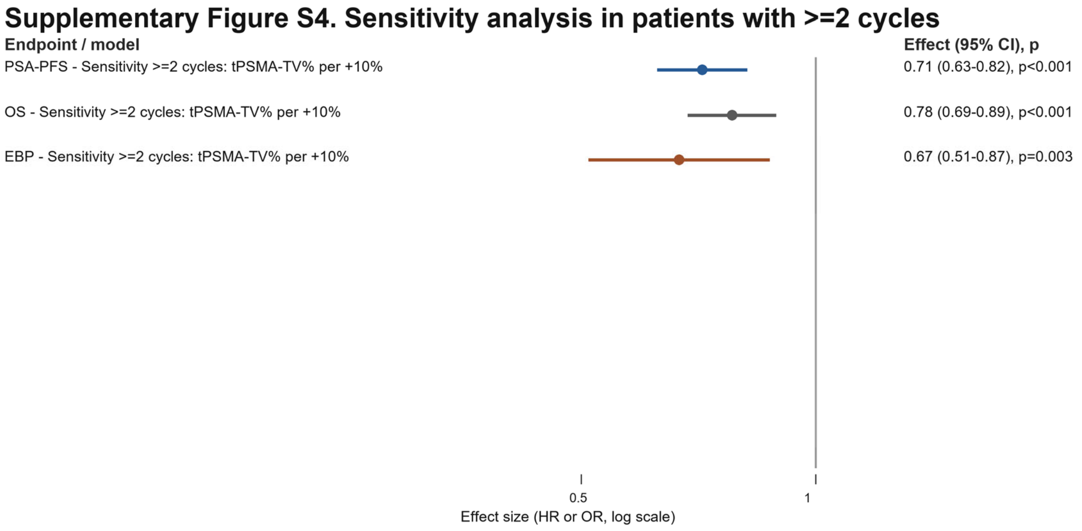

Figure S4. Sensitivity analysis restricted to patients completing at least two cycles, showing associations between tPSMA-TV% and PSA-PFS, OS, and EBP.

Abbreviations: CI, confidence interval; EBP, early biochemical progression; HR, hazard ratio; OR, odds ratio; OS, overall survival; PSA-PFS, prostate-specific antigen progression-free survival; tPSMA-TV%, percentage of high-uptake PSMA-positive tumor volume within total PSMA-positive tumor volume.

Figure S5. PSA Progression-Only Endpoint with Deaths Censored

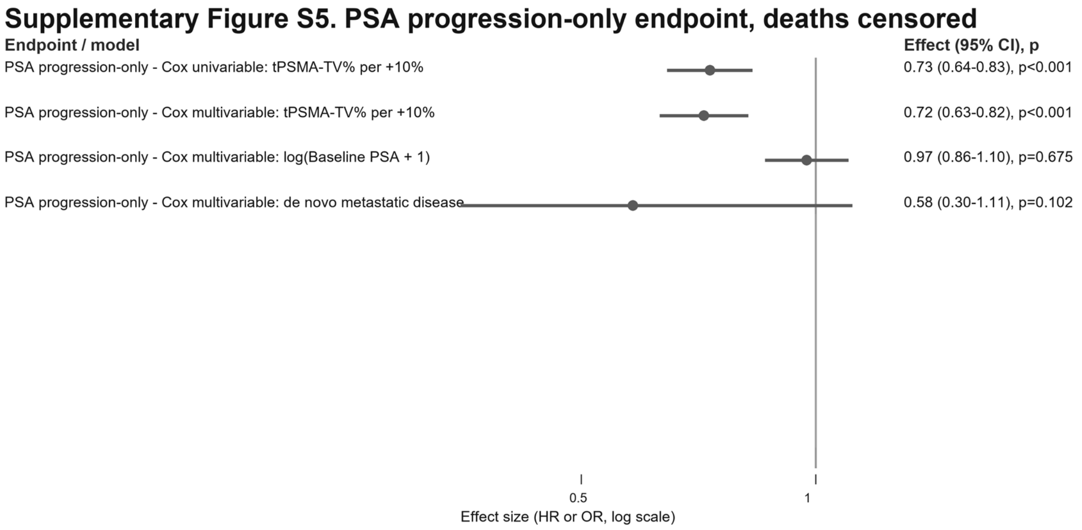

Figure S5. PSA progression-only endpoint analysis with deaths before documented PSA progression censored, showing univariable and multivariable Cox model estimates.

Abbreviations: CI, confidence interval; HR, hazard ratio; PSA, prostate-specific antigen; PSA-PFS, prostate-specific antigen progression-free survival; tPSMA-TV%, percentage of high-uptake PSMA-positive tumor volume within total PSMA-positive tumor volume.

Figure S6. Patient Flow Diagram

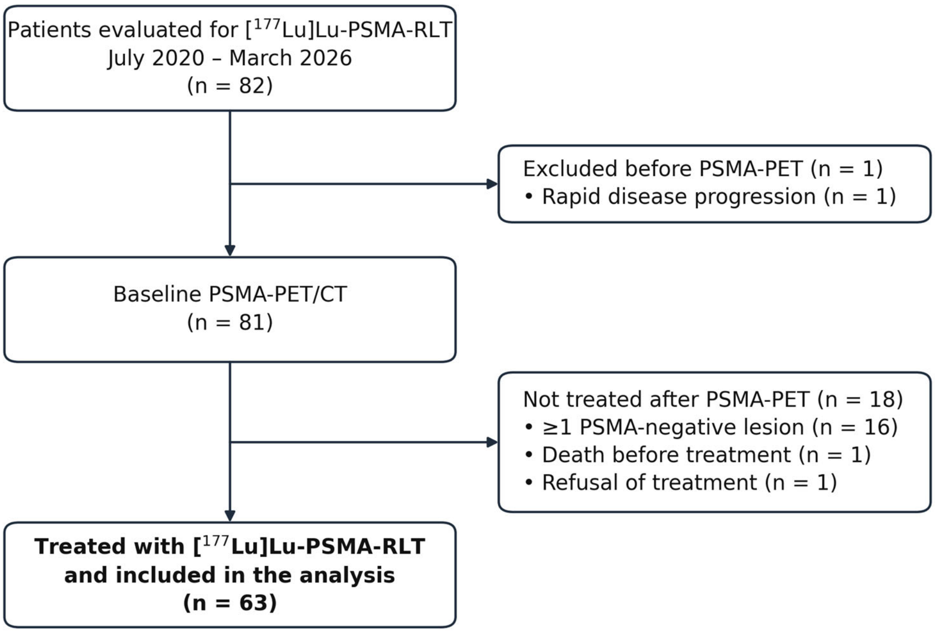

Figure S6. Patient flow diagram: 82 patients evaluated for [^{177}Lu]Lu-PSMA-RLT between July 2020 and March 2026, reasons for exclusion, and the 63 patients included in the analysis.
